# A Porous Hydrogel with High Mechanical Strength and Biocompatibility for Bone Tissue Engineering

**DOI:** 10.3390/jfb13030140

**Published:** 2022-09-03

**Authors:** Changxin Xiang, Xinyan Zhang, Jianan Zhang, Weiyi Chen, Xiaona Li, Xiaochun Wei, Pengcui Li

**Affiliations:** 1 College of Biomedical Engineering, Taiyuan University of Technology, Taiyuan 030024, China; 2 Shanxi Key Laboratory of Bone and Soft Tissue Injury Repair, Department of Orthopedics, The Second Hospital of Shanxi Medical University, Taiyuan 030001, China

**Keywords:** bone regeneration, hydrogels, mechanical properties, polyvinyl alcohol, tannic acid

## Abstract

Polyvinyl alcohol (PVA) hydrogels are considered to be ideal materials for tissue engineering due to their high water content, low frictional behavior, and good biocompatibility. However, their limited mechanical properties restrict them from being applied when repairing load-bearing tissue. Inspired by the composition of mussels, we fabricated polyvinyl alcohol/hydroxyapatite/tannic acid (PVA/HA/TA) hydrogels through a facile freeze–thawing method. The resulting composite hydrogels exhibited high moisture content, porous structures, and good mechanical properties. The compressive strength and tensile strength of PVA hydrogels were improved from 0.77 ± 0.11 MPa and 0.08 ± 0.01 MPa to approximately 3.69 ± 0.41 MPa and 0.43 ± 0.01 MPa, respectively, for the PVA/HA/1.5TA hydrogel. The toughness and the compressive elastic modulus of PVA/HA/1.5TA hydrogel also attained 0.86 ± 0.02 MJm^−3^ and 0.11 ± 0.02 MPa, which was approximately 11 times and 5 times higher than the PVA hydrogel, respectively. The PVA/HA/1.5TA hydrogel also exhibited fatigue resistance abilities. The mechanical properties of the composite hydrogels were improved through the introduction of TA. Furthermore, in vitro PVA/HA/1.5TA hydrogel showed excellent cytocompatibility by promoting cell proliferation in vitro. Scanning electron microscopy analysis indicated that PVA/HA/1.5TA hydrogels provided favorable circumstances for cell adhesion. The aforementioned results also indicate that the composite hydrogels had potential applications in bone tissue engineering, and this study provides a facile method to improve the mechanical properties of PVA hydrogel.

## 1. Introduction

Bone is the main weight-bearing tissue of the human body, and plays an essential role in supporting, protecting, transporting, and producing blood cells [1]. Specifically, tens of millions of people suffer from bone defects caused by trauma, developmental deformities, or tumor resection, which can influence their quality of life [2,3,4]. Natural bone can self-heal, to a certain extent, but large bone regeneration remains a major challenge, especially for load-bearing bone defects [5,6]. Although traditional autologous allografts are used clinically to repair large segments of bone defects, they still show various disadvantages, including a shortage of sources, additional trauma, need for a second surgery, and the high risk of disease transmission [6,7,8]. These complications motivate researchers to find suitable methods to repair defects. 

Tissue engineering is an alternative strategy that uses a combination of seed cells, biological factors, and scaffolds to repair or replace damaged tissues [9,10]. The ideal bone scaffolds should require suitable mechanical properties, inter-connectivity, porous structure, and excellent biocompatibility [11,12]. Among the optional material candidates, hydrogel plays a vital role in biomedical and biopharmaceutical treatments due to its resemblance to the extracellular matrix of natural tissues [13,14,15].

Polyvinyl alcohol (PVA) hydrogels have been widely used in tissue engineering due to their high water content, non-toxicity, and good biocompatibility [16]. However, the application of PVA hydrogels in bone tissue regeneration is restricted by their limited mechanical strength [17]. PVA hydrogels can be synthesized by physical and chemical methods. PVA hydrogels prepared by physical freeze–thawing methods can form crystal nuclei and hydrogen bonds to increase the crosslinked density of hydrogel networks, which can improve the mechanical properties [18]. Due to hydroxyapatite (HA) being one of the main components of bone tissue, it is often added in PVA hydrogels to mimic the structure and composition of natural bone tissue [19]. Recently, there have been significant efforts to design PVA/HA hydrogels for bone tissue engineering. HA was deposited in situ to fabricate the PVA/HA hydrogels with porous structures, but the compressive strength only attained 0.8 MPa [20]. A porous polyvinyl alcohol/sodium alginate/hydroxyapatite composite hydrogel with tunable structure and mechanical properties was fabricated by a dual-crosslinking method [16]. However, the pore size was about 1μm and the optimized compressive modulus was about 50 kPa. Hydroxypropyl guar was used to improve mechanical properties of PVA/HA hydrogels and the compressive strength the hydrogels attained was approximately 7 MPa [21], but the addition of HPG decreased the cell viability of MC3T3-E1 on HPG/PVA/n-HA. By utilizing in situ HA synthesis, the biocompatibility of PVA/HA hydrogels was enhanced [22]. However, there were no pores on the surface of the composite hydrogels, which hindered the nutrient and waste transportation of cells. Therefore, it is still a challenge to synthesize porous PVA/HA hydrogels with excellent mechanical properties and good biocompatibility.

Inspired by the structure and composition of mussels, catechol-based molecules, peptides, and polymers have been widely applied in tissue engineering [23]. For example, some researchers use the catechol groups in polydopamine (PDA) to form covalent/non-covalent bonds with different materials to modify the hydrogels [24]. However, their expensive price and dark color limit the practical applications of PDA. Tannic acid (TA) is moderate in color and cheaper than PDA. TA is a natural polyphenol, which is composed of pyrogallol and catechol groups [24]. TA can be crosslinked with other macromolecules through hydrogen bonds, ionic bonds, coordination bonds, and hydrophobic interactions [25,26,27]. The introduction of TA into PVA hydrogels was reported to improve the mechanical properties of hydrogels through hydrogen bonds [25,26,27].

In this study, PVA was used as the raw material to fabricate the composite hydrogels with the introduction of TA and HA into the PVA hydrogel using a facile physical method. The chemical and physical properties, microstructures, mechanical properties, and biocompatibility of PVA/HA/TA hydrogels were examined. Our study demonstrated that the introduction of TA and HA into the PVA hydrogel could improve mechanical strength and provide a suitable microenvironment for cell adhesion, proliferation, and composite hydrogels. These results showed potential applications in bone tissue engineering.

## 2. Materials and Methods

### 2.1. Materials

PVA-117 (Mw = 145,000) and TA (Aladdin Reagent Co., Ltd., Shanghai, China). Hydroxyapatite (Macklin Reagent Co., Ltd., Shanghai, China). Mouse pre-osteoblasts (MC3T3-E1) cells (Fenghui Biotechnology Co., Changsha, China), Fetal bovine serum (FBS), alpha minimum essential medium (α-MEM), and penicillin/streptomycin (Gibco, Invitrogen, Carlsbad, CA, USA). CCK8 kit and Live/Dead assay kit (Beyotime Biological Co. Ltd., Shanghai, China), Deionized water (UPR-IV-10 T, China).

### 2.2. Preparation of PVA/HA/TA Hydrogels

PVA (10 g, 10 wt%) and TA (0, 0.5 g, 1 g and 1.5 g, 0.5 wt%, 1 wt% and 1.5 wt%) were dissolved in 100 mL deionized water with the assistance of vigorous stirring for 3 h at 90 °C. Then, HA (5 g, 5 wt%) powders were added into the mixed solution and stirred to disperse homogeneously for 1 h at 90 °C. The final mixed solution was held for 1 h at room temperature to remove air bubbles. Next, the solution was poured into molds. Finally, the solution was frozen at −20 °C for 20 h and thawed at room temperature for 4 h. After five cycles of the freeze–thawing process, PVA/HA/TA hydrogels were prepared. These processes were the same as the synthesis of PVA hydrogel. The hydrogels with different contents of TA were named PVA, PVA/HA, PVA/HA/0.5TA, PVA/HA/1.0TA, and PVA/HA/1.5TA, respectively.

### 2.3. Fourier Transform Infrared Spectroscopy

Fourier transform infrared spectroscopy (FTIR) spectra of HA, PVA, TA, PVA/HA, and PVA/HA/0.5TA were obtained using an FTIR Spectrometer (Bruker, Alpha, Germany) in the transmittance mode within the range of 4000–450 cm^−1^ at a resolution of 4 cm^−1^ intervals, and the spectra plots represented 32 scans. Samples were mixed with KBr pellets to prepare the specimens for FTIR.

### 2.4. Scanning Electron Microscope

The microstructures of HA, PVA, PVA/HA, PVA/HA/0.5TA, PVA/HA/1TA, and PVA/HA/1.5TA hydrogels were observed by a scanning electron microscope (SEM, JEOL JSM-7100F, Japan) with an accelerating voltage of 5 kV. Before observation, the samples were freeze-dried and all samples were sputtered with platinum (Pt) prior to testing. The drying process consisted of putting the composite hydrogels into the liquid nitrogen for 5 min to pre-freeze before the pre-frozen hydrogels were moved into a freeze-dryer for 2 days of lyophilization.

### 2.5. Porosity

The porosity of the composite hydrogels was determined by a previous solvent immersion method, with slight modifications [28]. The freeze-dried hydrogels were cut into the same shape, weighed, and submerged into alcohol for 3 min. After taking out the samples, the extra alcohol was removed and the weight of the samples was measured. The porosity was calculated as follows:

$$\mathrm{Porosity}(\%)=\frac{W_{2}-W_{1}}{V\rho }\times 100\%$$

where W_2_ and W_1_ are the weight of the sample before and after immersion, respectively, V was the volume of the samples before immersion, and *ρ* was the density of alcohol. All tests were repeated 3 times.

### 2.6. Water Content

The water content of the composite hydrogels was calculated through the weight change of hydrogels before and after removing water. Filter paper was used to remove water from the surface of composite hydrogels before they were weighed. The moisture content was calculated using the followed equation:

$$\mathrm{Water}\;\mathrm{content}(\%)=\frac{W_{4}-W_{3}}{W_{4}}\times 100\%$$

W_4_ and W_3_ were the weight of the hydrogels before and after freeze-drying, respectively. All tests were repeated three times.

### 2.7. Mechanical Tests

The uniaxial tensile test was conducted on an electronic universal testing machine (Instron 5544, Boston, MA, USA) at room temperature. The samples for the compression tests were prepared in a cylinder shape (diameter of 15 mm, thickness of 10 mm) with a 2000 N sensor. The samples for tensile tests were prepared in a dumbbell shape (length of 30 mm, width of 4 mm, and thickness of 1 mm) with a 50 N sensor. The tensile strength and elongation of the hydrogels were measured at a strain rate of 50 mm·min^−1^ until breakage occurred. For the compression tests, the hydrogels were placed between the self-leveling plates and compressed at a rate of 5 mm·min^−1^ until the maximum strain reached 95%. The compressive elastic modulus was calculated at a slope range of 5–20% strain on the stress–strain curves. Toughness was obtained from the tensile stress–strain as the following equation:

$$\mathrm{Toughness}=\int_{0}^{\varepsilon_{\max}}\sigma d\varepsilon$$

where σ was the stress (MPa) and ε was the strain. For each sample, tests were measured at least 3 times and the average was reported.

Cyclic loading and unloading tests were also conducted on an electronic universal testing machine. The samples for the tests were prepared in the same way as the aforementioned samples. The rates of the tensile and compression tests were at 50 mm·min^−1^ and 5 mm·min^−1^, respectively. The cyclic loading−unloading test was performed to investigate the energy dissipation mechanism of the hydrogels. The dissipated energy was calculated as the following equation:

$$U_{\mathrm{hys}}=\int_{0}^{\varepsilon_{x}}\left(\sigma_{\mathrm{load}}-\sigma_{\mathrm{unload}}\right)d\varepsilon$$

here, σ_load_ and σ_unload_ were the loading and unloading stress. For each sample, tests were measured at least three times.

### 2.8. Cell Culture

The MC3T3-E1 cells were cultured in the alpha minimum essential medium (α-MEM, Gibco, Carlsbad, CA, USA), which contains 10% fetal bovine serum (FBS, Gibco, Carlsbad, CA, USA) and 1% penicillin-streptomycin (Gibco, Carlsbad, CA, USA) solution for the 5% CO_2_ incubator at 37 °C. The culture medium was refreshed every other day.

### 2.9. Cell Viability

All hydrogels were sterilized by autoclaving for one day and were washed by PBS three times before cell seeding. The as-prepared hydrogels were placed in a 24-well plate and were immersed in an α-MEM medium for 1 day. MC3T3-E1 was seeded onto the disk, PVA, PVA/HA, and PVA/HA/1.5TA at a density of 2 × 10^4^ cells per well. Then, cells were incubated for 1 or 5 days. A live/dead assay (Beyotime, Nantong, China) kit was used to determine the cytotoxicity of PVA/HA/1.5TA hydrogels. The medium was removed and the samples were washed with PBS twice, and the live/dead working solution was then added to each well. The live cells were stained with green, and dead cells were stained with red.

### 2.10. Cell Proliferation

A cell counting kit-8 (CCK-8, Beyotime, Nantong, China) was used to determine cell proliferation. The medium was removed and the samples were placed in a new 24-well culture plate after being washed with PBS twice. The 10% CCK-8 fresh culture medium was added to every well and incubated for 4 h in the dark at 37 °C. The optical density (OD) was measured by using a microplate reader (Biorad iMark, Hercules, CA, USA) at a wavelength of 450 nm.

### 2.11. Cell Morphology

The cell morphology on the hydrogels was observed by SEM. Cells were seeded on the 24 well-plates. After cultivating for 1 day, samples were rinsed with PBS three times and fixed with 2.5% glutaraldehyde. The samples were then dehydrated in sequence using a gradient series of ethanol (50%, 60%, 70%, 80%, 90% and 100%) for 10 min. The acquired samples were observed by SEM with platinum (Pt).

### 2.12. Statistical Analysis

All data were presented as mean ± standard error values, and analyzed using SPSS 14.0. A Student’s *t*-test or one-way ANOVA was used to evaluate the difference in means between different groups; *p* < 0.05 was considered statistically significant.

## 3. Results and Discussion

### 3.1. Preparation of PVA/HA/TA Hydrogels

**Figure 1:**
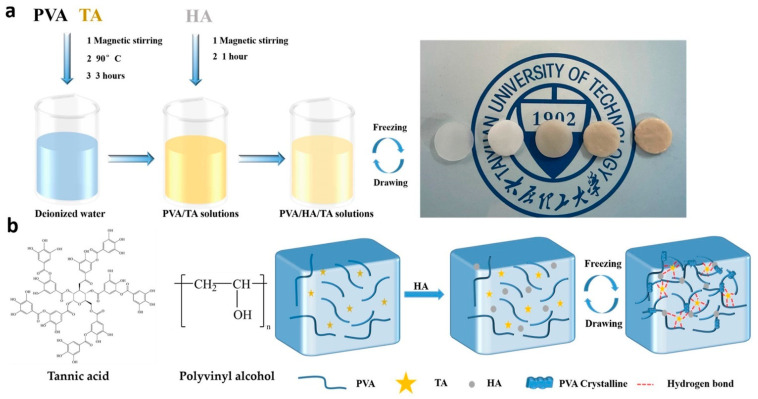
Schematic illustration of the preparation process (**a**) for the PVA/HA/TA hydrogel (from left to right, the hydrogels are PVA, PVA/HA, PVA/HA/0.5TA, PVA/HA/1.0TA, and PVA/HA/1.5TA) and the chemical structures (**b**) of the PVA/HA/TA hydrogel.

The preparation process of the composite hydrogels is briefly shown in Figure 1a. Figure 1b shows the chemical structures of the PVA and TA molecules. The TA molecule possessed a five-polyphenol-arm structure with 25 hydroxyl groups, which could form hydrogen bonds with hydroxyl groups on PVA and HA. As the contents of TA increased, the color of the hydrogels turned dark and opaque, as shown at Figure 1a. The hydroxyl groups between the PVA and TA molecules could form hydrogen bonds to build a robust physical crosslinked network. During the cyclic freeze–thawing processes, crystalline of PVA chains was also associated with multiple hydrogen bonds among HA, TA, and PVA molecules to densify the molecule network, as shown at Figure 1b. HA also served to mimic the natural bone structure and composition in PVA/HA/TA hydrogels. 

### 3.2. Characterization of PVA/HA/TA Hydrogels

#### 3.2.1. Fourier Transform Infrared Spectroscopy Analysis of Hydrogels

**Figure 2:**
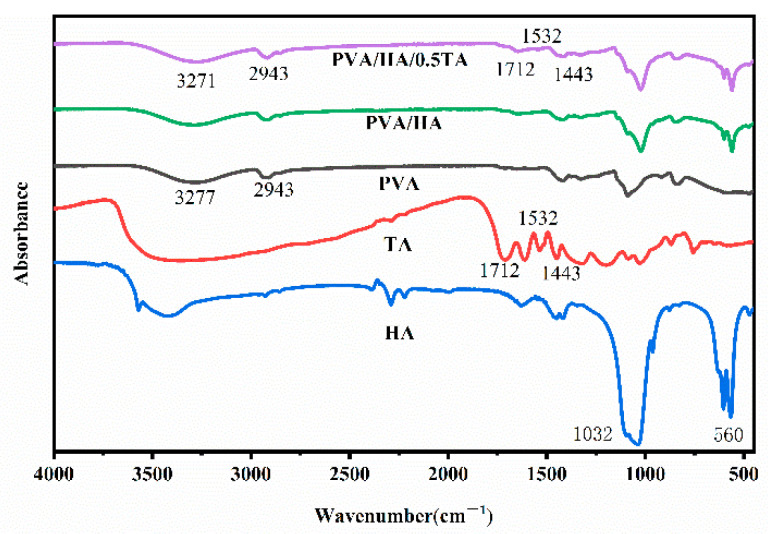
The FTIR spectra of PVA, HA, TA, PVA/HA, and PVA/HA/TA hydrogels.

To analyze the compositions and structural changes of the composite hydrogels, the pure PVA, HA, TA, PVA/HA, and PVA/HA/TA hydrogels were investigated by Fourier transform infrared spectroscopy (FTIR) analysis, as shown in Figure 2. The PVA exhibited characteristic absorption bands of -OH stretching vibrations at 3277 cm^−1^, -CH_2_ symmetric stretching vibrations at 2943 cm^−1^, and C-O stretching vibration at 1086 cm^−1^. The HA exhibited the characteristic absorption bands of PO_4_^3−^ at 1044 cm^−1^ and 962 cm^−1^. After the introduction of TA into the PVA/HA hydrogels, the absorption bands of the -OH stretching vibration shifted to lower wavenumbers at 3271 cm^−1^. According to previous studies, the formation of hydrogen bonds intra- and inter-molecule reduced the force constants of the -OH groups, which resulted in the shift of vibrational frequencies to lower wavenumbers [25,26,27]. The significant shifts of the absorption bands toward lower wavenumbers were due to the formation of hydrogen bonds when adding TA into the PVA/HA hydrogels. In addition, PVA/HA/TA hydrogels exhibited characteristic vibration peaks of TA at 1712 cm^−1^ (C=O), 1536 cm^−1^, and 1443 cm^−1^ (aromatic C-C), which implied the successful synthesis of PVA/HA/TA hydrogels via a facile and reliable method.

#### 3.2.2. Microstructure of Hydrogels

**Figure 3:**
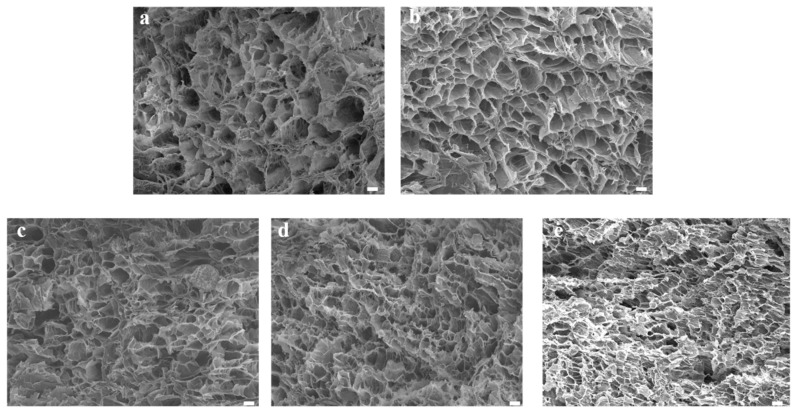
The SEM image of PVA (**a**), PVA/HA (**b**), PVA/HA/0.5TA (**c**), PVA/HA/1.0TA (**d**), and PVA/HA/1.5TA (**e**) hydrogels. Scale bars: 10 μm.

The microstructure of the hydrogels was characterized by scanning electron microscopy (SEM). As illustrated in Figure 3, the microstructures of the PVA, PVA/HA, PVA/HA/0.5TA, PVA/HA/1.0TA, and PVA/HA/1.5TA hydrogels presented interconnected porous structures. The interconnected microstructures of the composite hydrogels were beneficial for cell attachment and proliferation, which could promote new bone formation. Meanwhile, the numerous pore structures provide abundant space for drug diffusion and release [29]. After adding the HA, the microstructure of PVA hydrogels remained unchanged and the distribution of the pores was still uniform. Compared with PVA and PVA/HA hydrogels, the pore size of PVA/HA/TA hydrogels became smaller due to the dense crosslinked network. When the content of TA attained 1.5%, continuous pores still existed, but the diameter of the pores was reduced to less than 10μm. The TA molecules might serve as new crosslinked points to form a dense crosslinked network [30].

#### 3.2.3. Porosity and Water Content of Hydrogels

**Figure 4:**
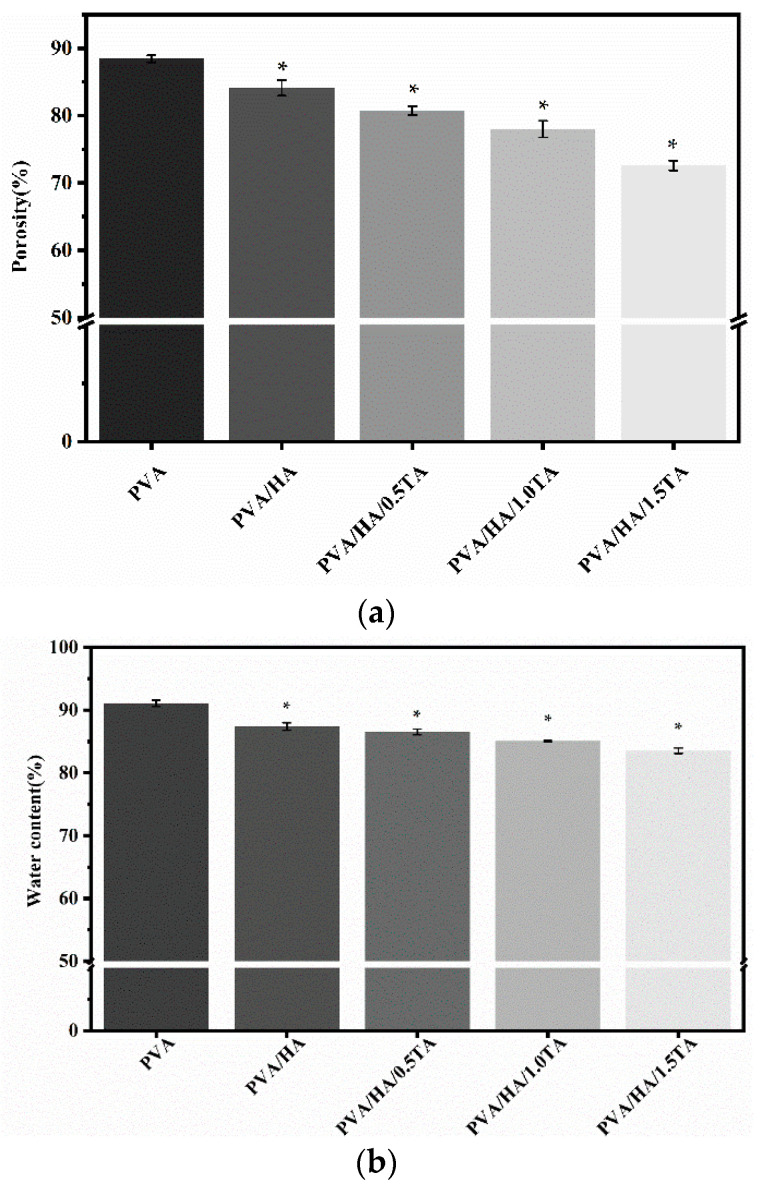
The porosity (**a**) and water content (**b**) of PVA, PVA/HA, PVA/HA/0.5TA, PVA/HA/1.0TA, and PVA/HA/1.5TA hydrogels. * *p* < 0.05 compared to PVA group.

The porosities of the PVA, PVA/HA, PVA/HA/0.5TA, PVA/HA/1.0TA, and PVA/HA/1.5TA hydrogels were 88.4 ± 0.5%, 84.1 ± 1.2%, 80.7 ± 0.6%, 78.0 ± 1.2%, and 72.6 ± 0.7%, respectively (Figure 4a). The newly formed hydrogen bonds resulted in a significant decrease in the porosity ratio of the composite hydrogels (*p* < 0.05). However, appropriate porosity was beneficial for cell proliferation and nutrient transportation [29], and the high porosity provided abundant space for drug diffusion and controlled release [30]. Previous studies have demonstrated that suitable porosity (70%) of hydrogel scaffolds is required to create new bone tissue [31].

The water content was an important characteristic for hydrogels as tissue regenerative materials, which directly influenced the nutrient transport [32]. As shown in Figure 4b, the water contents of the PVA, PVA/HA, PVA/HA/0.5TA, PVA/HA/1.0TA, and PVA/HA/1.5TA hydrogels were 91.1 ± 0.4%, 87.4 ± 0.5%, 86.5 ± 0.5%, 85.1 ± 0.1%, and 83.5 ± 0.4%, respectively. The water content of the composite hydrogels decreased significantly with the increase in TA content (*p* < 0.05). PVA contained many hydroxyl groups and it was easy to form hydrogen bonds with TA, which resulted in a lower water content, though the water content of the PVA/HA/1.5TA hydrogels still attained 83.51%.

#### 3.2.4. Mechanical Properties of Hydrogels

**Figure 5:**
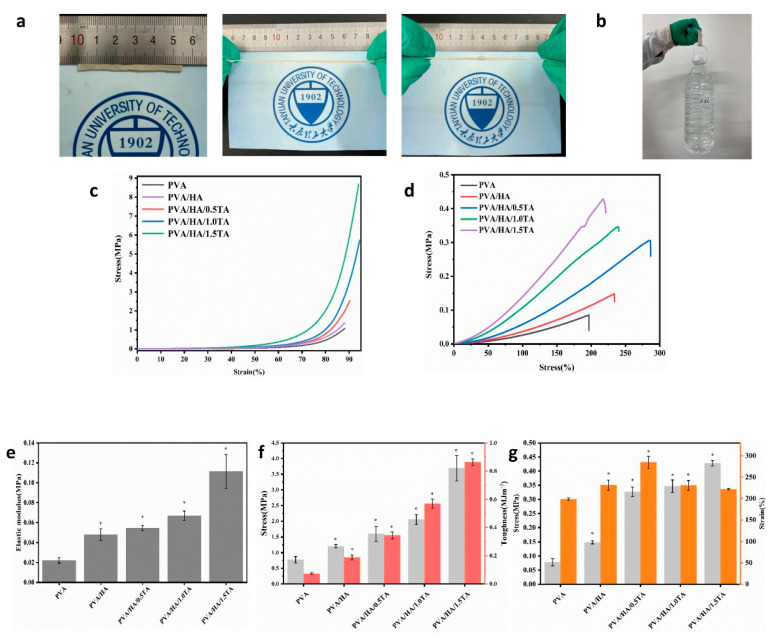
Photographs of PVA/HA/TA hydrogel stretching (**a**) while withstanding a weight of 2 L water. (**b**,**c**) Compressive curves of the PVA, PVA/HA, PVA/HA/0.5TA, PVA/HA/1.0TA, and PVA/HA/1.5TA hydrogels. (**d**) Tensile curves of the PVA, PVA/HA, PVA/HA/0.5TA, PVA/HA/1.0TA, and PVA/HA/1.5TA hydrogels. (**e**) Elastic modulus of PVA, PVA/HA, PVA/HA/0.5TA, PVA/HA/1.0TA, and PVA/HA/1.5TA hydrogels. (**f**) Compressive stress and toughness of PVA, PVA/HA, PVA/HA/0.5TA, PVA/HA/1.0TA, and PVA/HA/1.5TA hydrogels. (**g**) Tensile strength and breakage elongation of PVA, PVA/HA, PVA/HA/0.5TA, PVA/HA/1.0TA, and PVA/HA/1.5TA hydrogels. * *p* < 0.05 compared to the PVA group.

An ideal hydrogel for tissue engineering should satisfy the mechanical requirements of different tissues. Excellent mechanical performance is a necessary requirement for hydrogels applied in bone-tissue engineering. Therefore, a series of mechanical experiments were carried out with composite hydrogels. The PVA/HA/1.5TA hydrogel was able to bear bending and knotting and could withstand compression to a large strain without the occurrence of damage (Figure 5a). The PVA/HA/1.5TA hydrogel could load with approximately 2.0 L water (Figure 5b). The tensile and compressive stress–strain curves of the composite hydrogels are presented in Figure 5c,d. The mechanical performances of composite hydrogels were improved after the introduction of TA. The compressive strengths of the PVA, PVA/HA, PVA/HA/0.5TA, PVA/HA/1.0TA, and PVA/HA/1.5TA hydrogels were 0.77 ± 0.11 MPa, 1.20 ± 0.05 MPa, 1.60 ± 0.23 MPa, 2.05 ± 0.16 MPa, and 3.69 ± 0.41 MPa, respectively. The compressive elastic modulus of the PVA/HA/1.5TA hydrogels was 0.111 ± 0.17 MPa, which was five times higher than the PVA hydrogels (*p* < 0.05). The tensile strength of the PVA hydrogel was only 0.08 ± 0.01 MPa, but the tensile strength of PVA/HA/1.5TA attained 0.42 ± 0.01 MPa after the addition of TA. The fracture toughness also significantly increased from 0.07 ± 0.01 MJm^−3^ for the PVA hydrogel to 0.86 ± 0.02 MJm^−3^ for PVA/HA/1.5TA (*p* < 0.05). In a previous study, a biomimetic porous hydrogel was fabricated using interactions between amino hydroxyapatite and gelatin/gellan gum, the compressive stress of this hydrogel at 80% strain only attained approximately 0.7 MPa [33]. When the PVA was solely combined with HA, the mechanical properties of the PVA/HA hydrogel were only slightly improved. Therefore, the introduction of TA was favorable for the improvement of mechanical strength and toughness. The improvements of the tensile and compressive performance of the composite hydrogels were due to the numerous hydrogen bonds that formed between the abundant hydroxyl groups of PVA, HA, and TA. In comparison to previous studies, a nanomaterial was introduced into the PVA hydrogels to improve their mechanical properties, and the compressive strength was only 600 kPa [34]. By adding a magnesium oxide nanoparticle and black phosphorus nanosheet into polyvinyl alcohol/chitosan hydrogel, a multifunctional hydrogel was fabricated to repair the bone defects. However, the compressive strength and elastic modulus of the hydrogels only attained 70 kPa and 3 kPa, respectively [35]. The PVA/HA/TA hydrogels in our study not only exhibited porous structures and high water contents, but also exhibited excellent mechanical properties. The strain of PVA/HA/0.5TA was 248.6 ± 0.1% and the strain of PVA/HA/1.5TA was 221.5 ± 2.0% (Figure 5g).

**Figure 6:**
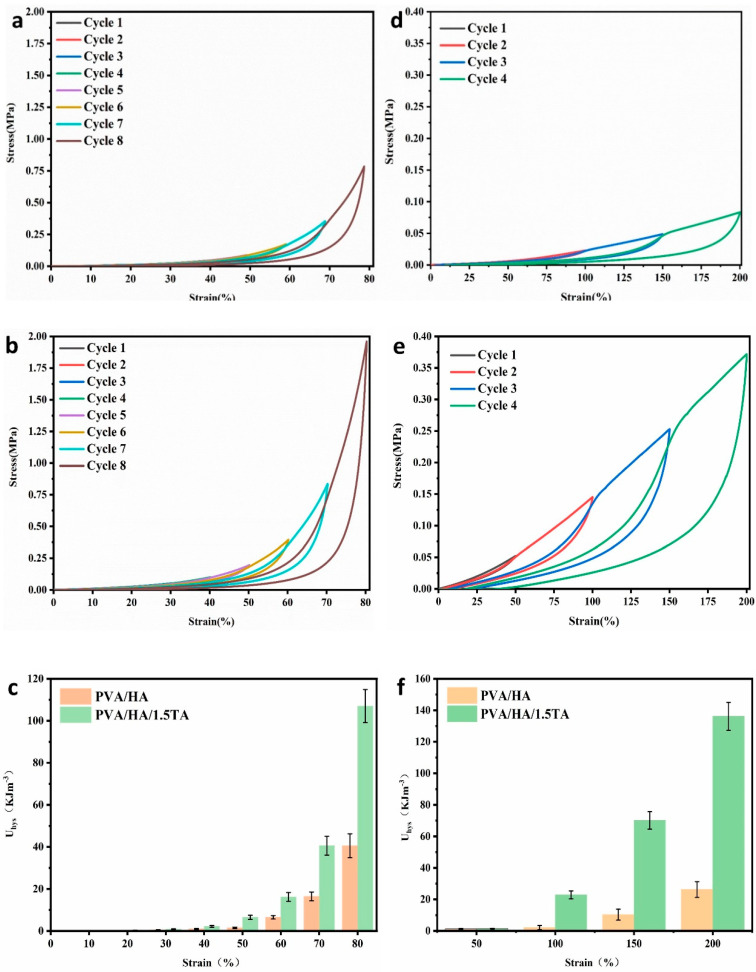
(**a**) Loading–unloading compressive test and tensile test of PVA/HA (**a**,**d**) and PVA/HA/1.5TA (**b**,**e**) under different strain values and the calculated dissipated energy (curve area) (**c**,**f**) of the PVA/HA and PVA/HA/1.5TA hydrogels during the loading–unloading cycles with different strains.

The energy dissipation ability of the hydrogels influenced their practical applications [36]. Hydrogen bonds were able to store and dissipate the energy effectively to improve the mechanical properties of composite hydrogels [37]. In this study, the PVA/HA hydrogel and PVA/HA/1.5TA hydrogel were chosen to confirm the dissipation ability of hydrogels at varying strains. As shown in Figure 6, the PVA/HA/1.5TA hydrogel presented pronounced hysteresis loops during the loading–unloading processes. The PVA/HA/1.5TA hydrogel showed more dissipated energy than the PVA/HA hydrogel did. The dissipated energy of the PVA/HA/1.5TA hydrogel increased two times and four times, respectively, in comparison to the PVA/HA hydrogel at 80% compressive strain and at 200% tensile strain; this may be due to richer hydrogen bonds in the PVA/HA/1.5TA hydrogel. Previous studies have demonstrated that the strong hydrogen bonds could serve as permanent crosslink points, while weak hydrogen bonds could break and reconstruct to dissipate the energy in PVA/TA hydrogels during the loading–unloading cycles [26].

**Figure 7:**
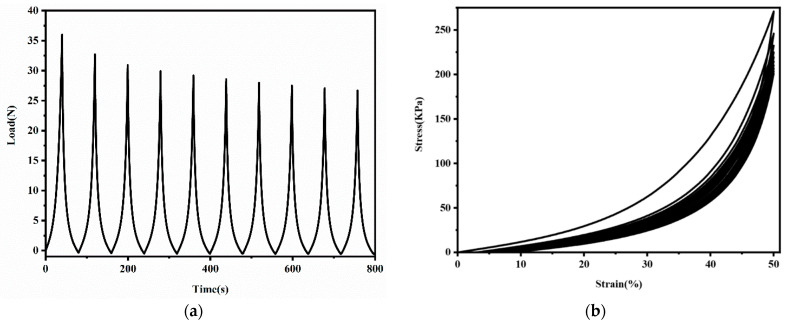
The load–time curve (**a**) and stress–strain curve (**b**) of ten successive compression–relaxation cycles of the PVA/HA/1.5TA hydrogel.

For application as bone-tissue materials, hydrogels were required to bear repetitive force for a short period of time. In this study, ten cyclic loading–unloading compressive tests were performed on the PVA/HA/1.5TA hydrogel at fixed strains to evaluate its stability. As shown in Figure 7, the maximum force decreased slightly after every cycle in the force–time curve and the residual strains remained small in the stress–strain curve. The stress–strain of PVA/HA/1.5TA hydrogel nearly coincide from the third to tenth loading cycles. After ten successive cycles, the maximum stress still remained 74.1% of the initial stress. The PVA/HA/1.5TA hydrogels exhibited excellent energy dissipation abilities and good mechanical stability. The physical crosslinked network may dynamically break and reconstruct during the loading–unloading processes.

### 3.3. Biological Properties of Hydrogels

#### 3.3.1. Cell Viability and Proliferation

**Figure 8:**
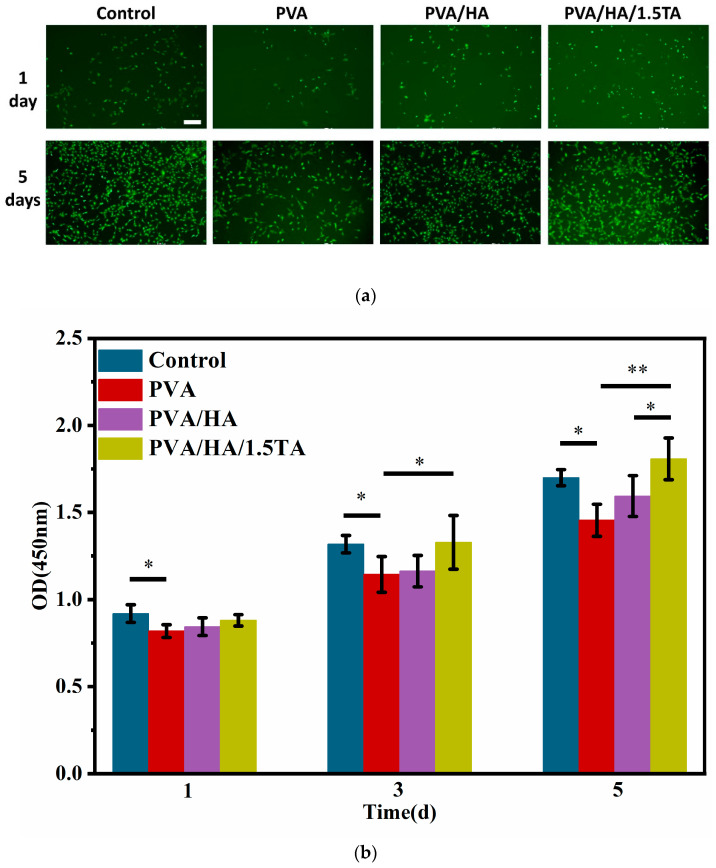
Cell viability and proliferation of MC3T3-E1 cells. (**a**) Live/dead assay for the viability of MC3T3-E1 cells on different hydrogels for 1 day and 5 days. (**b**) CCK8 assays for the proliferation of MC3T3-E1 cells on different hydrogels for 1, 3, and 5 day(s). (* *p* < 0.05, ** *p* < 0.01). Scale bar: 200 um.

The PVA/HA/1.5TA hydrogel exhibited excellent mechanical properties with high water content and porosity, so it was chosen for cell experiments. Cell viability was evaluated by using live/dead staining assays at 1 and 5 days. (Figure 8a). All hydrogels were shown to be nontoxic to cells with few dead cells during the culture period. The density of the cells on the hydrogels increased with culture time. Compared to the PVA hydrogel, more cells were observed on PVA/HA/1.5TA hydrogel, which indicates that the TA and HA enhanced cell viability. Cell proliferation on the different hydrogels was assessed by CCK-8 proliferation assays at 1, 3, and 5 days. According to Figure 8b, the CCK-8 absorption of cells on each hydrogel increased with culture time, which indicates that the PVA/HA/1.5TA hydrogel did not affect the MC3T3-E1 proliferation. The cell proliferation of the control group was higher than the PVA group at 1 day (*p* < 0.05). During the experimental groups, the PVA/HA/1.5TA was shown to have higher proliferation than the PVA group at 3 days (*p* < 0.05). After 5 days, the absorbance values of the PVA/HA/1.5TA composite hydrogel were higher than the PVA and PVA/HA hydrogels (*p* < 0.05). PVA/HA/1.5TA hydrogel could promote cell proliferation better than PVA hydrogel. The aforementioned results proved that the biocompatibility of the PVA/HA/1.5TA hydrogel is good.

#### 3.3.2. Cell Morphology

**Figure 9:**
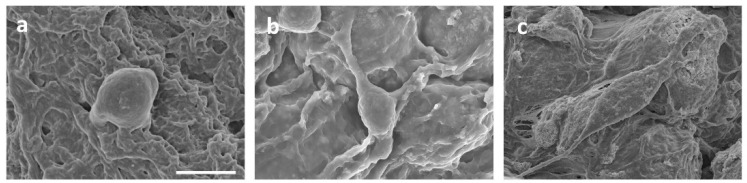
Cell morphologies of MC3T3-E1 on the PVA (**a**), PVA/HA (**b**), and PVA/HA/1.5TA (**c**) hydrogels. Scale bar: 10 um.

SEM images presented how the different hydrogels affected the spread of MC3T3-E1 cells (Figure 9). The cells spread on the surface of the PVA/HA/1.5TA hydrogels with obvious pseudopodia for 1 day. In contrast, cells on the PVA and PVA/HA hydrogels were not fully extended, which indicates that the PVA/HA/1.5TA hydrogels could provide a better environment for MC3T3-E1 cell adhesion than PVA and PVA/HA hydrogels. The PVA exhibited a low adhesion ability for cells due to the hydrophilic materials [38,39]. On the PVA/HA hydrogel, we observed that the cells were attached and had begun to spread. After the introduction of TA, it could be seen that there was more cell adhesion. Previous studies showed that bone-cells tended to grow on rigid substrates [20]. Therefore, the PVA/HA/1.5TA hydrogels might provide a suitable microenvironment for MC3T3-E1 cell growth due to their excellent mechanical properties and proper porosity.

## 4. Conclusions

In this study, a new composite hydrogel with high water content, porous structures, excellent mechanical properties, and good biocompatibility were fabricated via a facile physical method. The high porosity and porous structure of the PVA/HA/TA hydrogels are beneficial for cell nutrient and waste transport. The microstructures and mechanical properties of the composite hydrogels could be flexibly adjusted by adjusting the content of TA. In addition, the PVA/HA/TA hydrogels were shown to be more favorable for cell proliferation, spreading, and adhesion. This study shows the potential applications of composite hydrogels in bone tissue engineering. However, the mechanical properties of these composite hydrogels were still lower than natural bone, and the biofunctions of composite hydrogels in vivo also need to be evaluated in further studies.

## Data Availability

The data generated from the study is clearly presented and discussed in the manuscript.
